# Comparing N-mixture models and GLMMs for relative abundance estimation in a citizen science dataset

**DOI:** 10.1038/s41598-022-16368-z

**Published:** 2022-07-19

**Authors:** Benjamin R. Goldstein, Perry de Valpine

**Affiliations:** Department of Environmental Science, Policy, and Management, University of California, Berkeley, Berkeley, CA 94720 USA

**Keywords:** Ecology, Ecological modelling, Computational models, Software, Statistical methods

## Abstract

To analyze species count data when detection is imperfect, ecologists need models to estimate relative abundance in the presence of unknown sources of heterogeneity. Two candidate models are generalized linear mixed models (GLMMs) and hierarchical N-mixture models. GLMMs are computationally robust but do not explicitly separate detection from abundance patterns. N-mixture models separately estimate detection and abundance via a latent state but are sensitive to violations in assumptions and subject to practical estimation issues. When one can assume that detection is not systematically confounded with ecological patterns of interest, these two models can be viewed as sharing a heuristic framework for relative abundance estimation. Model selection can then determine which predicts observed counts best, for example by AIC. We compared four N-mixture model variants and two GLMM variants for predicting bird counts in local subsets of a citizen science dataset, eBird, based on model selection and goodness-of-fit measures. We found that both GLMMs and N-mixture models—especially N-mixtures with beta-binomial detection submodels—were supported in a moderate number of datasets, suggesting that both tools are useful and that relative fit is context-dependent. We provide faster software implementations of N-mixture likelihood calculations and a reparameterization to interpret unstable estimates for N-mixture models.

## Introduction

Understanding how species’ abundances are associated with covariates of interest is a primary goal of species distribution modeling^1^. Often the best one can hope to estimate are patterns of *relative abundance*, sometimes referred to as an index of abundance. Relative abundance values are equal to absolute abundance—the number of individuals in a known area—multiplied by an unknown constant such as a detection rate and/or the inverse of effective area sampled. When the unknown constant can be assumed to be the same between two areas, the ratio of relative abundances is the same as the ratio of absolute abundances, allowing comparison of sites relative to each other. Relative abundance can sometimes be estimated from data when absolute abundance cannot, but doing so can be challenging when data are collected with heterogeneous sampling protocols because variation in the data collection process can obscure or confound patterns in abundance. Large, heterogeneous datasets are becoming more prominent in ecology, such as those produced by citizen science^2,3^, autonomous recorders^4,5^, and camera traps^6^. Analyzing these data requires statistical models that fit the data well, account for details of study design and sampling if possible, and can be estimated efficiently.

Two existing model types can satisfy this requirement: generalized linear mixed models (GLMMs) and N-mixture models. The GLMM is a linear model extension that models relationships between non-Gaussian (e.g. count) response data while allowing hierarchical structure via random effects. To estimate relative abundance, count data are modeled as Poisson-distributed with an expected value defined by a log-linked linear combination of important covariate data, and a link-scale random effect is added to account for relatedness between replicate observations at a site^7^. The GLMM is a heuristic model developed to explain patterns but does not correspond to a data generating process for repeated counts of unknown, finite numbers of individuals.

In contrast to the GLMM, the N-mixture model’s development was motivated by process-based thinking, and its architecture corresponds to an idealized data generating process^8^. In the N-mixture model, a latent state *N* represents the absolute abundance of a species at a sampling site. *N* varies between sites according to a Poisson distribution with expected value log-linked to linear covariates and is assumed to be constant across replicate observations at a site (the “closure” assumption). The observed data are binomial distributed with size *N* and probability *p*, representing the detection process, such that variation in counts within a site is due only to observer error^8–10^. While all within-site variation is considered observation error and all between-site variation stems from the underlying abundance process, in practice heterogeneous observation error and movement of animals can lead to detection-driven variation between sites and abundance-driven variation within sites.

Though mathematically distinct, both GLMMs and N-mixture models partition variation into between- and within-site components using hierarchical relationships. Between sites, the N-mixture has a Poisson random latent abundance at each site, while the GLMM has a log-scale normal random effect at each site. Within sites, the N-mixture model uses binomial counts, while the GLMM uses Poisson counts. Each model has variants that accommodate overdispersion in counts: in the GLMM, a negative binomial distribution may replace the Poisson, while in the N-mixture model both the Poisson and binomial may be replaced with a negative binomial or beta-binomial distribution, respectively, to account for overdispersion in the within- and/or between-site submodel^8,11^. However, in the N-mixture model, the latter variants can be highly computationally demanding, so one contribution here is a set of more efficient ways to calculate likelihoods for these variants. More detailed descriptions of both models are presented in the “Model implementation and selection” section of the Methods.

These models exist within a shared heuristic framework in that both models have parameters that predict “number of individuals observed” at a standardized location and primarily differ in their assumptions of between- and within-site variation in observed counts. It is important to distinguish between estimation of parameters and estimation of latent states, such as the latent state *N* in the N-mixture model. Parameter estimation and model selection arise from making a model do as well as possible as a probability distribution for observed data, not latent states. In other words, both models predict “how many individuals will I see.” Users of N-mixture models are often interested in the follow-on question of “how many individuals are there, adjusted for imperfect detection,” but that is an interpretation of parameters and latent states outside of fitting criteria. Hence, the models can be compared based on their fit to observed data. Although “all models are wrong,” knowing which model fits the data best is useful for characterizing patterns.

Within this heuristic framework, both the GLMM and N-mixture model come with advantages and disadvantages. The main advantage in using a GLMM is that it is robust to unmodeled heterogeneity in data generation due to its relatively simple structure. Robustness to unmodeled variation is a useful quality when analyzing count data with unobserved heterogeneity in sampling protocol or skill, unknown and heterogeneous sampling areas, or complicated non-independence between observations. The primary drawback to using the GLMM is that this model does not explicitly account for the detection process. In fact, GLMMs only estimate relative abundance under the assumption that the pattern of interest is not confounded with detection after accounting for other modeled variables^12^. When this assumption is unreasonable, the GLMM may not be useful for understanding biological patterns, even when it fits the data best.

The N-mixture model, in contrast, explicitly separates abundance from imperfect detection, but at the cost of strong sensitivity to violations in model assumptions, especially those assumptions related to an absence of unmodeled heterogeneity (see Supplemental Section 1 for an enumeration of N-mixture modeling assumptions). In recent years, simulation studies have characterized the degree to which N-mixture models produce biased or nonsensical estimates of abundance in the presence of unmodeled heterogeneity^11,13–17^. Additionally, Kéry^19^ identified estimation instability, likely attributable to a likelihood maximum in the limit of zero detection^18^, but recommended the use of N-mixture models when estimation instability does not occur^19^. Several studies have found that the N-mixture model produces estimates of absolute abundance that agree with more rigorous sampling methods^20–23^ though this finding is not universal^24^. Still, established sensitivities and computational pathologies are grounds for caution in recommending N-mixture models when data do not strictly conform to modeling assumptions.

In this paper, we provide guidance for choosing between the GLMM and N-mixture model for relative abundance estimation in an empirical context. We ask which of a set of GLMM and N-mixture model variants best fits single-species subsets of eBird point-count data on a small spatial scale. eBird, the largest and most systematic citizen science data repository of its kind, is increasingly used to estimate bird species’ spatiotemporal distributions^25,26^. Because eBird data inherently contain unobserved heterogeneity, they present an interesting challenge for both GLMMs and N-mixture models (see Supplement 1).

eBird citizen scientists report their observations in the form of “checklists”, lists of detected species associated with sampling metadata. Almost 90% of eBird checklists are “complete checklists”, which imply zero counts for all unreported species. While much statistical modeling of eBird data has addressed estimation over large spatial extents^27–29^, a simpler yet still challenging goal is to estimate local abundance patterns using data from regions with concentrated replicate observations. We select 396 species-subregion (SSR) subsets of eBird across gradients of space, checklist density, and species abundance. Within each SSR, we use only eBird checklists from stationary sampling locations (i.e. checklists obtained from a single spatial point) to have the highest chance of satisfying N-mixture model assumptions.

We consider four variants of the N-mixture model and two of the GLMM. Each N-mixture variant is defined by the two distributions in the within- and between-site submodels, and we consider four variants: the classic binomial-Poisson (B-P), binomial-negative binomial (B-NB), beta-binomial-Poisson (BB-P) and beta-binomial-negative binomial (BB-NB). In the GLMM, we consider the traditional Poisson distribution for counts alongside the negative binomial to allow for overdispersion. We fit each model variant to each dataset with step-wise variable selection. To characterize relative fit across models, we use the Akaike information criterion (AIC) because it is derived to select the model with lowest out-of-sample prediction error^30^. We explore patterns in selection across levels of abundance and sampling intensity and apply a suite of goodness-of-fit and estimation checks to characterize known issues with both the N-mixture and GLMM. We investigate the special issue of estimation instability with a reparameterization of the N-mixture abundance and detection intercepts. We also implement new, fast algorithms for calculating N-mixture likelihoods for variants accommodating overdispersion.

## Results

### Species-subregion (SSR) datasets

Our procedure for aggregating “subregions” yielded 20 circular subregions of 10 km radii containing the highest density of eBird activity in California during the 2019 breeding season. (Supplemental Fig. 1; see Supplemental Section 2 for the full SSR selection algorithm). Subregions corresponded to human population centers with access to natural habitat such as large parks or coastal areas. Subregions contained between 140 and 1000 high quality checklists distributed across 12 to 140 unique locations. In each subregion, we selected 10 species with high detection rates and 10 species with intermediate detection rates. We further selected 10 species overall with sufficient data in the most total subregions to compare model selection for individual species across space. In all, we compared models of interest across 396 species-subregion (SSR) subsets of eBird.

### Model selection

**Figure 1:**
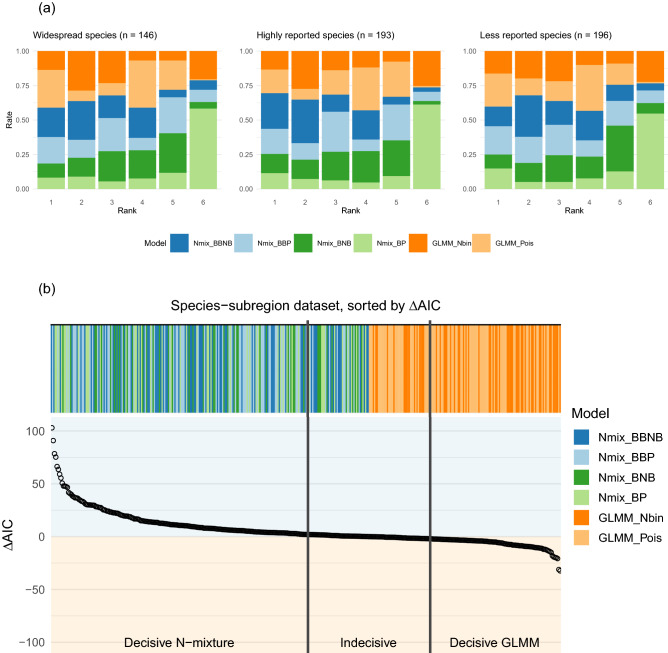
(**a**) Model rankings by species reporting rate category (1 = best). (**b**) Magnitude of AIC difference between the best N-mixture model and the best GLMM for each dataset. Each stripe-point pair represents one SSR dataset. Bar color indicates AIC model choice; point position on the y-axis indicates the difference in AIC ($\Delta$AIC) between the best N-mixture model and the best GLMM.

We fit each of six N-mixture and GLMM variations to each SSR dataset. Across SSRs, the six models we considered were chosen by AIC at the following rates: Poisson GLMMs were selected in 88 datasets (22%), negative binomial GLMMs in 61 datasets (15%), BB-NB N-mixture models in 79 datasets (20%), BB-P N-mixture models in 74 datasets (19%), B-NB N-mixtures in 46 datasets (12%) and B-P N-mixtures in 48 datasets (12%) (Fig. 1). AIC clearly selected ($\Delta$AIC > 2) the best GLMM over the best N-mixture model in 102 (26%) datasets, while the best N-mixture model was selected in 199 (50%). AIC rankings indicated overall support for models incorporating overdispersion.

Negative binomial GLMMs outperformed Poisson GLMMs in 308 of 396 datasets. However, they were largely superseded in those cases by N-mixture models, especially those that included a beta-binomial-distributed detection submodel (BB-P and BB-NB N-mixture models). Among N-mixture variants, the best N-mixture model by AIC used a beta-binomial distribution in 223 datasets (56%), and these cases largely corresponded to cases where GLMMs also outperformed binomial-submodel N-mixture models (B-P and B-NB). When beta-binomial N-mixtures were excluded from the analysis, GLMMs were dominant, being selected clearly by AIC ($\Delta$AIC > 2) in 237 (60%) datasets.

**Figure 2:**
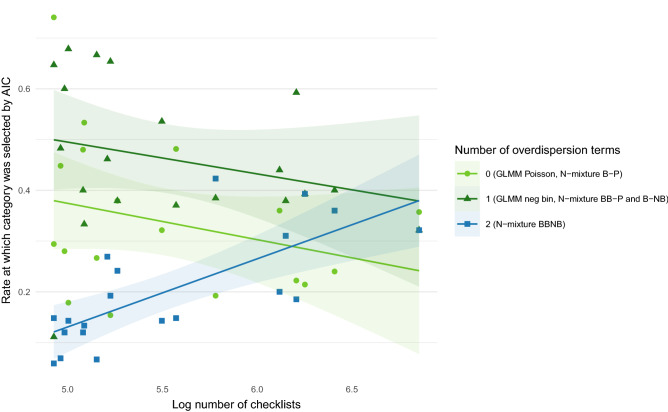
For each subregion, we plot the proportion of each model group selected for each of three model flexibility categories against the number of checklists in that subregion. Solid lines show best fit lines for each category. As the number of checklists in a subregion is increased, the most flexible BB-NB N-mixture model is supported at an increasing rate.

Subregions with more checklists were associated with higher rates of selection of the most complicated model (by number of overdispersion parameters), the BB-NB N-mixture model (Fig. 2). We attribute this to the phenomenon that more data contains more information to be explained by additional model structure.

We did not detect patterns in model selection related to whether a species was overall highly detected or detected at intermediate rates (Fig. 1). We did not identify patterns in model selection varying by species identity among widespread species (Supplemental Fig. 3).

Eleven SSR datasets were removed from the analysis after modeling due to computational issues with GLMM estimation (see Supplemental Section 5).

### Fit, estimation, and computation

#### Goodness-of-fit

**Figure 3:**
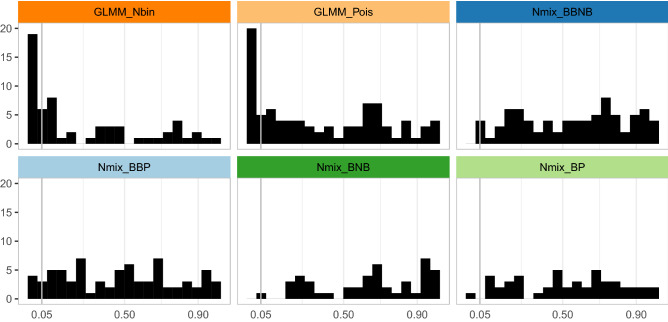
Goodness-of-fit *p*-values for N-mixture models selected by AIC had near-uniform distributions, while a subset of selected GLMMs showed goodness-of-fit failures.

We tested goodness-of-fit (GOF) from residuals for each model and assessed systematic patterns of fit by examining the distributions of GOF *p*-values for each model type. Among N-mixture models, distributions of goodness-of-fit *p*-values did not deviate from the uniform, meaning that most N-mixture models selected by AIC fit well for this metric. Both sets of selected GLMMs showed deviation from a uniform distribution of *p*-values, indicating that these models’ residuals deviated meaningfully from modeling assumptions for some datasets (Fig. 3). GLMMs were selected for several datasets where GOF checks for GLMMs failed, despite those same datasets passing goodness-of-fit checks for N-mixture models, indicating that AIC model selection did not correspond exactly to goodness-of-fit metrics (Supplemental Fig. 4).

None of the models considered incorporated spatial autocorrelation. We tested that assumption using Moran’s I tests on residuals for all models. Nine percent of model-dataset combinations had *p*-values less than 0.05 from Moran’s I test, indicating more cases of nonrandom spatial structure in residuals than expected by chance. This suggested the presence of spatial autocorrelation in some datasets, but at a low rate we considered acceptable for this study’s conclusions.

#### Parameter estimation

**Figure 4:**
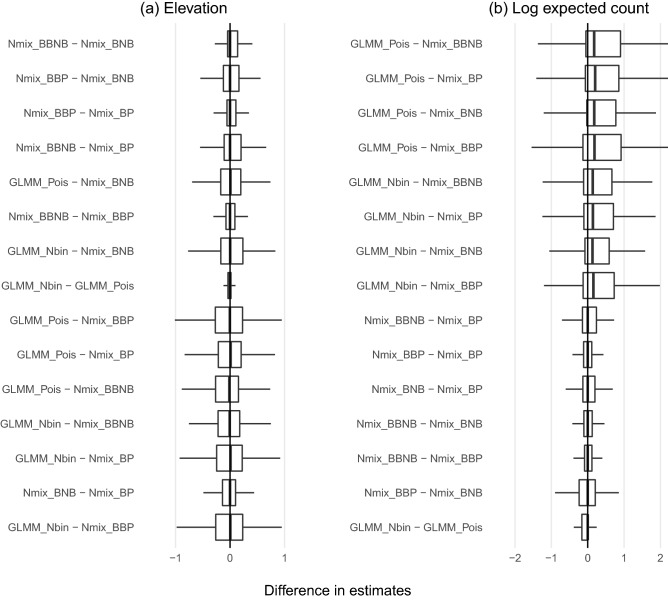
Distributions of the absolute differences between models in (**a**) log-scale effect of elevation and (**b**) log expected count. Model pairs are ordered by median difference. Outliers are excluded for legibility. Log expected count is the log-scale intercept plus 0.5 times the random effects variance for the GLMMs and simply the log-scale expected count intercept for N-mixture models. The latter is defined in our parameterization as a combination of abundance and detection intercepts.

Point estimates of the log expected count at the mean site (i.e. with all centered covariates set to 0), adjusted for the log-normal random effects of the GLMM, were similar for GLMMs and N-mixture models (Fig. 4). Within model type, N-mixture models agreed closely with one another, as did GLMMs. Differences in a point estimate of a coefficient, site elevation, were centered around zero for all model combinations, indicating no systematic differences between or within model types.

**Figure 5:**
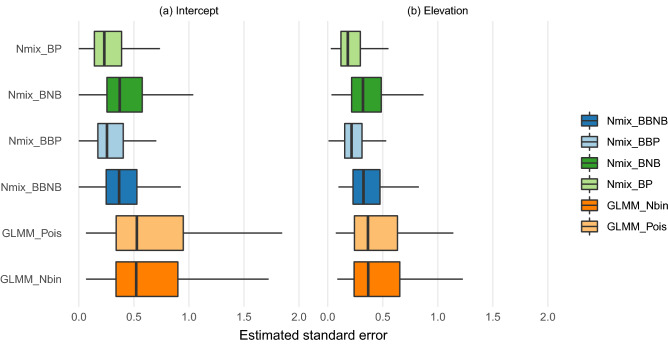
Comparing standard errors of estimates of the data intercept (defined as expected log count at a standard site) and a relative abundance driver of interest, elevation. Outliers are excluded for legibility.

Estimated standard errors of covariates were systematically different between models (Fig. 5). Both GLMMs estimated standard errors systematically larger than all N-mixture models, while more complex N-mixture models estimated larger standard errors (as expected within a model family).

#### Stability of parameter estimates

We investigated rates of instability in N-mixture estimation by monitoring whether estimated AIC and two intercept parameters changed as the upper bound of the truncated infinite sum, *K*, was increased^19^. Across 396 species-region datasets, 6% of B-P N-mixture models, 7% of BB-P models, 37% of B-NB models, and 11% of BB-NB models were found to be unstable in AIC for a tolerance of $\Delta$AIC = 0.1. While the B-NB models showed by far the highest rate of instability, agreeing with previous findings by Kéry^19^, some instability was detected across all N-mixture models. To illustrate that instability could be attributed to indeterminate tradeoff between detection and abundance, we reparameterized the two intercepts with one parameter for (intercept of) log observed count (abundance $\times$ detection) and another for (intercept of) log ratio between detection and abundance (abundance / detection). The log observed count intercept was stable in 93–98% of datasets in each of the four N-mixture models. The log ratio intercept, representing tradeoff between detection and abundance, was unstable in patterns mirroring those of AIC. This suggested that instability with increasing K was due to ridged likelihoods in a single parameter direction that was a combination of the original parameters.

#### Performance of the fast N-mixture calculation

We extended previous work by Meehan et al.^45^ to implement an algorithm for fast N-mixture likelihood calculations (see Supplemental Section 3). Fast N-mixture calculations led to 20- to 90-fold improvements in computation time compared to the naive method for calculating the truncated infinite sum for $100 \le K \le 2000$ and $5 \le \text{ length }(y_i) \le 20$, where $y_i$ was the vector of detection-nondetection observations at a site (Supplemental Fig. 5). These gains increased with $\text{ length }(y_i)$, such that the contribution of the fast algorithm was more important when more replicate visits were made to a simulated site. In eBird, sites are clustered such that a small number of locations contain many replicate observations, making this improvement per replicate particularly relevant. Improvements in absolute computation time were largest for the beta-binomial and negative binomial N-mixture variants, which take orders of magnitude longer than the traditional B-P N-mixture due to their higher computational costs.

## Discussion

When estimating relative abundance from count data, ecologists can consider both N-mixture models and GLMMs alongside one another in a model selection context. Both N-mixture models and GLMMs act as predictors of counts that vary between and within sites and can be compared via standard model comparison tools. By adopting a heuristic perspective towards models based on predictive fit, we let the data speak about which model is better rather than guessing *a priori* based on assumptions about the data-generating process. Put another way, we accept that all models under consideration are “wrong” in that assumptions are bound not to be perfectly satisfied. By selecting among the candidate models with AIC, we learn which model approximately minimizes out-of-sample prediction error.

Across 396 species-subregion data subsets of eBird, we found that it was usually possible to distinguish between N-mixture and GLMM fit and that N-mixture models outperformed GLMMs somewhat more often than the reverse. This pattern was contingent on the inclusion of beta-binomial N-mixtures, which greatly outperformed binomial N-mixture variants. All four N-mixture model variants also more consistently passed goodness-of-fit checks. Consideration of only one model type across all these data would produce worse overall fit than dataset-by-dataset selection.

We identified no patterns in model selection across species characteristics or site identity, indicating that relative fit was highly context-dependent. We observed one pattern in model selection: datasets containing more checklists (and therefore more information) were more likely to select more complex models. This trend does not suggest that more complex models like the BB-NB N-mixture model were “true” for our data, and that it was therefore wrong to use a simpler model for low-information datasets, since in fact simpler models minimize out-of-sample prediction error in lower-information contexts. The overall lack of patterning suggests that ecologists should not assume that a particular model will fit better for a given dataset.

Point estimates of a parameter of interest, effect of elevation on count, agreed between N-mixture models and GLMMs, but GLMMs estimated systematically larger standard errors than N-mixture models. In general, lower standard errors are not reason for recommending a model, because lower standard errors can result from either correctly characterized improvements in precision, or overconfidence (and increased Type I error); without access to true data generating parameters these are indistinguishable without additional fit metrics. If both models fit reasonably well, then agreement between parameters may indicate support for the assumption that elevation was not confounded with detection. While most selected N-mixture models passed goodness-of-fit checks, a substantial portion of GLMMs chosen by AIC failed them, indicating that GLMMs fit the data somewhat more poorly overall. A simulation study targeting goodness-of-fit and model selection patterns across known data-generating conditions could clarify whether this relatively poorer fit is due to actual better correspondence between N-mixture models and the eBird data generating process, or whether N-mixture models are more flexible when data are heterogeneous in general. Comparing intercepts between the models (expected log count at a “typical” site) is more complicated due to their different structures. Specifically, the expected count from the GLMM’s log-normal intercept distribution needs adjustment by the random effects variance to be comparable to the corresponding N-mixture parameter. After this adjustment, we see that log expected counts are not systematically higher or lower for one kind of model (see Supplemental Section 6).

Estimation instabilities in N-mixture models were largely attributable to a single dimension representing the decomposition between abundance and detection, while parameter estimates of relative abundance drivers were estimated stably. These instabilities correspond to a boundary estimate of detection probability being nearly zero. Although this is implausible in the mechanistic motivation of N-mixture models, from a statistical perspective a boundary estimate is not necessarily a pathological estimation outcome, so these unstable cases can still be compared alongside other models. We found a high rate of instability in the B-NB N-mixture model in agreement with Kéry^19^, but found that this high rate did not extend to the BB-NB and BB-P N-mixture model variants, which showed rates of instability comparable to the traditional B-P N-mixture. Still, instability was present at low rates in all four N-mixture variants. Future theoretical work may clarify how to interpret this phenomenon when it occurs.

We analyzed datasets from a single important database (eBird), during a single year, and at modest spatial scales, so the particular patterns we observed in model selection are limited. We also chose an aggressive data filtering approach that brought the data closer into alignment with N-mixture modeling assumptions. It is possible that N-mixture models would be less successful on less aggressively filtered data. For feasibility, we chose to only consider two model families of interest. One relevant linear model extension, the generalized additive mixed model (GAMM), was excluded from this analysis. GAMMs can be used to introduce flexibility into parameters which are allowed to vary over space and/or time^31,32^. Because of the potential for estimating spatially variable covariate effects, ecologists working at larger spatial scales may want to consider GAMMs alongside N-mixture models and GLMMs where appropriate. In this application, we expected that this flexibility would not be relevant at the spatial scale considered. Neither GLMMs nor N-mixture models overwhelmingly outperformed one another by AIC, but it is conceivable that new models could be developed to extend or bridge these approaches.

When choosing between GLMMs and N-mixture models for relative abundance estimation, practitioners may weigh trade-offs beyond the models’ different abilities to explain the data (as characterized by an information criterion and goodness-of-fit checks). When an abundance covariate of interest is confounded with detection, using an N-mixture model may disentangle these two components, while using a GLMM will not. If estimating absolute abundance is of interest, the GLMM will similarly not satisfy this need, while the N-mixture model could if effective area sampled is known and standard assumptions are satisfied. Practitioners interested in either of these two approaches should consider modeling assumptions during study design and data collection. On the other hand, the GLMM may be preferred when computational efficiency is important, such as when the number of observations is very large. The marginalized likelihoods for N-mixture variants presented in this paper reduce the computational costs of N-mixture model fitting. Fast implementations of the N-mixture likelihood calculation reduced computation times 20- to 90-fold (see Supplemental Section 3, Supplemental Fig. 5). These implementations are available in the R package nimbleEcology^33^.

We found that observed instability with increasing *K*^14,19^ was not prohibitive for interpreting relative abundance estimates. We used a parameterization that suggests such instability arises from a parameter estimate on the boundary of the parameter space, which is a manageable and not uncommon problem in other kinds of models. Relative abundance point estimates agreed between N-mixture models and equivalently constructed GLMMs. This suggests that practitioners should not reject the N-mixture model in the presence of this form of estimation instability, and can consider both N-mixture models and GLMMs when estimating relative abundance with count data. Without access to known truth, we could not investigate whether the estimates produced reflect true relationships between covariates and species abundance, so we cannot say whether either N-mixture models or GLMMs estimated parameters accurately in that sense. We suggest that practitioners interested in modeling relative abundance from count data consider the assumptions of both models, including known features of N-mixture model robustness in practice and theory^10,11,13,14,16–18,20–23^, along with other practical trade-offs. If both GLMMs and N-mixture models are potentially appropriate, we recommend the model selection and goodness-of-fit checking procedure outlined in this paper for choosing the most parsimonious model with adequate fit.

To estimate species’ relative abundance patterns from increasingly common heterogeneous datasets, ecologists will find both N-mixture models and GLMMs useful on a context-dependent basis. Despite their distinct origins in heuristic and process-based thinking, these two models are structurally analogous tools for predicting counts that vary both across and within sampling units. We have presented results to indicate that selecting between these models for a particular dataset is possible on a context-dependent basis. We encourage ecologists to adopt a holistic approach to model selection, considering different models alongside one another and bearing in mind that statistical models, whether or not they are process-motivated by design, are ultimately tools for fitting data.

## Methods

All figures were produced using the R package ggplot2 v3.3.5^34^.

### eBird and covariate data

eBird data are structured as follows. Birders submit observations as species checklists with counts of each species they identify. They report associated metadata, such as location, date and time, duration of the observation period, number of observers, and sampling protocol^25,26,31^. The birder indicates whether their checklist is “complete”; complete checklists yield inferred zeroes for all species not reported on a checklist.

We retrieved the eBird Basic Dataset containing all eBird observations and sampling metadata. We extracted all complete checklists that occurred within the U.S. state of California between April 1 and June 30, 2019. Four survey-level covariates were retrieved from eBird checklist metadata as detection covariates: number of observers, checklist duration, date of year, and time of day; any checklist that failed to report one or more of these variables was dropped. Corresponding to best practices for use of eBird data, we filtered the data for quality according to the following criteria: we discarded checklists other than those following the “Stationary” survey protocol (observations made at a single spatial location) with duration shorter than 4 hours and at most 10 observers in the group^31,35^.

We selected twenty circular regions of high sampling intensity with 10 km radii across California (Supplemental Fig. 1). These spanned the state’s many habitats including coastal, agricultural, wetland, and mountain areas, and contained active birding areas such as parks and human population centers. In each subregion, we selected 10 species with the highest reporting rate (proportion of checklists including that species) and 10 representing an intermediate reporting rate. An additional 10 species were selected that were detected in many regions to enable cross-region comparisons, yielding 407 species-subregion (SSR) datasets (with overlaps between the two species selection protocols; see Supplemental Section 2 for the full algorithm). Across 20 subregions, we accepted 6094 eBird checklists for analysis, each with an associated count (potentially zero) for each species. Observations were aggregated to sampling sites defined by a 50 m spatial grid. The 50 m grid was chosen to conservatively identify related surveys and was not motivated by biological processes, nor does it represent the sampling area of each survey. In this context, the concept of “closure” in the latent state is already suspect due to the fact that eBird checklist sampling areas are inconsistent. Data were processed in R using the ‘auk’ package^36,37^.

An elevation surface for the state of California was retrieved from WorldClim at $8.3 \times 10^{-3}$ decimal degrees resolution using the R package raster^38,39^. This commonly used covariate was included as a baseline spatial covariate to enable comparison of estimation properties across sites, but its biological relevance to abundance is not crucial to our analysis^31^. Land cover data were retrieved from the LandFire GIS database’s Existing Vegetation Type layer^40^. For each unique survey location, a 500 m buffer was calculated around the reported location, and the percent of the buffer which was water, tree cover, agriculture or other vegetation (shrub or grassland) was calculated. We used the following five site-level covariates: elevation, and percent of the landscape within a 500 m buffer of the site that was water, trees, agricultural land, or other vegetation. We included six checklist-level covariates: duration, number of observers, time of day, time of day squared, Julian date, and Julian date squared. Covariates were dropped in datasets where only a single unique value was observed for that covariate.

### Model implementation and selection

We considered four variants of the N-mixture model and two variants of the GLMM comprising a total of 6 distinct models, defined by the distributions used in the model or sub-model.

The GLMM for count data that we considered is defined as

$$\begin{array}{*{20}l} {y_{{ij}} \sim D(\mu _{{ij}} ,[\theta ])} \hfill \\ {\log (\mu _{{ij}} ) = \beta _{0} + {\mathbf{x}}_{{ij}}^{T} \user2{\beta } + \alpha _{i} } \hfill \\ {\alpha _{i} \sim {\mathcal{N}}(0,\sigma _{\alpha } )} \hfill \\ \end{array}$$

where $y_{ij}$ is th *j*th observation at site *i*, *D* is a probability distribution (which may contain an extra parameter $\theta$ to account for overdispersion), $\mu _{ij}$ represents the mean expected count and is a logit-linear combination of observed site- and observation-level covariates $x_{ij}$, $\beta$ are coefficients representing the effect of those covariates, $\beta _0$ is a log-scale intercept corresponding to the expected log count at the mean site (i.e. with all centered covariates set to 0), and $\alpha _i$ is the random effect of site *i* following a normal distribution. Due to the right skew of $\exp (y_{ij})$, by log-normal distribution theory the log of the expected count at the mean site is $\beta _0 + 0.5 \sigma _{\alpha }^2$. We considered two forms of this model, where *D* was either a Poisson or a negative binomial distribution, in the latter case with the extra parameter $\theta$.

The N-mixture model is defined as

$$\begin{array}{*{20}l} {y_{{ij}} \sim D_{w} (N_{i} ,p_{{ij}} ,[\theta _{w} ])} \hfill \\ {N_{i} \sim D_{b} (\lambda _{i} ,[\theta _{b} ])} \hfill \\ {{\text{logit}}(p_{{ij}} ) = {\text{}}{\text{logit}}(p_{0} ) + {\mathbf{x}}_{{ij(w)}} {\mathbf{\beta }}_{w} } \hfill \\ {\log (\lambda _{i} ) = \log (\lambda _{0} ) + {\mathbf{x}}_{{i(b)}} {\mathbf{\beta }}_{b} } \hfill \\ {p_{0} = e^{{\frac{{\phi _{1} + \phi _{2} }}{2}}} } \hfill \\ {\lambda _{0} = e^{{\frac{{\phi _{1} - \phi _{2} }}{2}}} } \hfill \\ \end{array}$$

where $D_b$ and $D_w$ are probability distributions representing between- and within-site variation, respectively; $N_i$ is a site-level latent variable normally representing the “true” abundance at site *i*; $p_{ij}$ is the detection probability of each individual on the *j*th observation event at site *i*; $\lambda _i$ is the mean abundance at site *i*; and $x_{(w)}$ and $x_{(b)}$ are covariate vectors for detection and abundance, respectively, with corresponding coefficients $\beta _w$ and $\beta _b$. For reasons described below, we reparameterize the intercept parameters of the N-mixture submodels, $\log (\lambda _0)$ and $\text{ logit }(p_0)$, in terms of two orthogonal parameters $\phi _1 = \log (\lambda _0 p_0)$ and $\phi _2=\log (p_0 / \lambda _0)$. Now $\phi _1$ and $\phi _2$ represent the expected log count and the contrast between detection and abundance, respectively, at the mean site. This parameterization allows us to investigate stability of parameter estimation. The log-scale expected count of the N-mixture model is $\phi _1 = \log (\lambda _0 p_0)$, analogous to $\beta _0 + 0.5 \sigma _{\alpha }^2$ in the GLMM (see Supplemental Section 6). Each submodel distribution *D* could include or not include an overdispersion parameter ($\theta _w$ and $\theta _b$), yielding four possible N-mixture model variants: binomial-Poisson (B-P), binomial-negative binomial (B-NB), beta-binomial-Poisson (BB-P), and beta-binomial-negative binomial (BB-NB)^8,11^.

We chose to fit models with maximum likelihood estimation (MLE) for computational feasibility and because key diagnostic tools, such as AIC and methods for checking goodness-of-fit and autocorrelation, were best suited to MLE estimation^15^. We fit N-mixture models with the nimble and nimbleEcology R packages starting with a conservatively large choice of K, the truncation value of the infinite sum in the N-mixture likelihood calculation^33,41^ (see Supplemental Section 4 for a discussion of maximum likelihood estimation with NIMBLE). We fit GLMMs with the R package glmmTMB^42^. We applied forward AIC selection to choose the best covariates for each model with each dataset (illustrated in Fig. S1). One spatial covariate (elevation) and two checklist metadata covariates (duration and number of observers) were treated as *a priori* important and were included in all models. In the N-mixture model, checklist-specific sampling metadata were only allowed in the detection submodel, while land cover covariates and the interactions between them were allowed in both the detection and abundance submodels. Interactions were dropped in datasets when interaction values showed a correlation of > 0.8 with one of their first-order terms. In N-mixture models, additions to both submodels were considered simultaneously during forward AIC selection.

For comparisons between models, we selected a heuristic threshold of $\Delta \text {AIC}> 2$ to say that one model is supported over another^30^.

### Fit, estimation, and computation

#### Goodness-of-fit

We used the Kolmogorov-Smirnov (KS) test, a *p*-value based metric, to evaluate goodness-of-fit on each selected model. For GLMMs, residuals were obtained using the DHARMa R package’s ‘simulateResiduals’ and the KS test was applied using the ‘testUniformity’ function^43^. For N-mixture models, we considered the site-sum randomized quantile (SSRQ) residuals described by Knape et al.^15^, computing these for each N-mixture model and running a KS test against the normal CDF. We assumed that covariate effects did not vary by space within subregions and chose not to use spatially explicit models^31,44^. To test this assumption, we applied Moran’s I test to the SSRQ or DHARMa-generated residuals for each site or observation.

#### Parameter estimation

We compared two abundance parameters of interest across models: coefficients for elevation and log expected count at a standard site (in the GLMM, $\beta _0 + 0.5 \sigma _\alpha ^2$; in the N-mixture model, $\log (\lambda _0 p_0)$). We examined absolute differences in point estimates and the log-scale ratios between their standard errors.

#### Stability of estimated parameters

Attempting to decompose the expected value of observed data into within- and between-site components can lead to ridged likelihood surfaces with difficult-to-estimate optima. Kéry found that instability of model estimates with increasing K occurred when there was a likelihood tradeoff between detection and abundance, resulting in a tendency in abundance toward positive infinity restrained only by K^10^. Dennis et al. showed that N-mixture models could in fact yield estimates of absolute abundance at infinity^18^. We interpreted this as a case of a boundary parameter estimate rather than non-identifiability and explored it by reparametrizing as follows. We estimated the intercepts for detection and abundance with two orthogonal parameters (rotated in log space) $\phi _1 = \log (\lambda _0 p_0)$ and $\phi _2 = \log (p_0 / \lambda _0)$, where $\lambda _0$ and $p_0$ are real-scale abundance and detection probability at the mean site. We hypothesized that in unstable cases, $\phi _1$, log expected count, is well-informed by the data, but $\phi _2$, the contrast between abundance and detection, is not well-informed, corresponding to a likelihood ridge as $\phi _2 \rightarrow -\infty$ due to detection probability approaching 0 and abundance approaching infinity. This reparameterization isolates the likelihood ridge to one parameter direction, similar to a boundary estimate as $\exp (\phi _2) \rightarrow 0$. Boundary estimates occur in many models and are distinct from non-identifiability in that they result from particular datasets. Confidence regions extending from a boundary estimate may include reasonable parameters, reflecting that there is information in the data. We defined a practical lower bound for $\phi _2$. When $\phi _2$ was estimated very near that bound, we conditioned on that boundary for $\phi _2$ when estimating confidence regions for other parameters.

In the N-mixture case, diagnosing a boundary estimate for $\phi _2$ is made more difficult by the need to increase K for large negative $\phi _2$ to calculate the likelihood accurately. We used an approach like that of Dennis et al.^18^ to numerically diagnose unstable cases. For each N-mixture variant in each SSR, the final model was refitted twice, using values of K 2000 and 4000 greater than the initial choice. Estimates were considered unstable if the absolute value of the difference in AIC between these two large-K refits was above a tolerance of 0.1. We monitored whether MLE estimates of $\phi _1$ and $\phi _2$ also varied with increasing K.

#### Evaluating the fast N-mixture calculation

We extended previous work by Meehan et al. to drastically improve the efficiency of N-mixture models using negative binomial or beta-binomial distributions in submodels^45^ (see Supplemental Section 3).

We ran benchmarks of this likelihood calculation for a single site against the traditional algorithm, which involves iterating over values of *N* to compute a truncated infinite sum. We calculated the N-mixture likelihood at 5,000 sites and compared the computation time between the two methods for all four N-mixture model variations. We ran benchmarks along gradients of $\text {length}(y_i)$ (number of replicate observations at the simulated site) and K (the upper bound of the truncated infinite sum) for each variant.

## Supplementary Information

Supplementary Information.

## Data Availability

All analyses were performed using publicly available data.

## Code availability

All code is available in a GitHub repository.

## References

[1] Elith J, Leathwick JR (2009). Species distribution models: ecological explanation and prediction across space and time. Annu. Rev. Ecol. Evol. Syst..

[2] Chandler M (2017). Contribution of citizen science towards international biodiversity monitoring. Biol. Cons..

[3] Silvertown J (2009). A new dawn for citizen science. Trends Ecol. Evol..

[4] Furnas BJ, Callas RL (2015). Using automated recorders and occupancy models to monitor common forest birds across a large geographic region: automated recorders monitoring common birds. J. Wildl. Manag..

[5] Kahl S, Wood CM, Eibl M, Klinck H (2021). BirdNET: a deep learning solution for avian diversity monitoring. Eco. Inform..

[6] Steenweg R (2017). Scaling-up camera traps: monitoring the planet’s biodiversity with networks of remote sensors. Front. Ecol. Environ..

[7] Bolker BM (2009). Generalized linear mixed models: a practical guide for ecology and evolution. Trends Ecol. Evol..

[8] Royle JA (2004). N-mixture models for estimating population size from spatially replicated counts. Biometrics.

[9] Royle JA, Dorazio RM (2006). Hierarchical models of animal abundance and occurrence. J. Agric. Biol. Environ. Stat..

[10] Kéry M, Royle JA (2016). Applied Hierarchical Modeling in Ecology.

[11] Martin J (2011). Accounting for non-independent detection when estimating abundance of organisms with a Bayesian approach: correlated behaviour and abundance. Methods Ecol. Evol..

[12] Dénes FV, Silveira LF, Beissinger SR (2015). Estimating abundance of unmarked animal populations: accounting for imperfect detection and other sources of zero inflation. Methods Ecol. Evol..

[13] Barker RJ, Schofield MR, Link WA, Sauer JR (2018). On the reliability of N-mixture models for count data. Biometrics.

[14] Duarte A, Adams MJ, Peterson JT (2018). Fitting N-mixture models to count data with unmodeled heterogeneity: bias, diagnostics, and alternative approaches. Ecol. Model..

[15] Knape J (2018). Sensitivity of binomial N-mixture models to overdispersion: the importance of assessing model fit. Methods Ecol. Evol..

[16] Link WA, Schofield MR, Barker RJ, Sauer JR (2018). On the robustness of N-mixture models. Ecology.

[17] Monroe AP, Wann GT, Aldridge CL, Coates PS (2019). The importance of simulation assumptions when evaluating detectability in population models. Ecosphere.

[18] Dennis EB, Morgan BJ, Ridout MS (2015). Computational aspects of N-mixture models: computational aspects of N-mixture models. Biometrics.

[19] Kéry M (2018). Identifiability in N -mixture models: a large-scale screening test with bird data. Ecology.

[20] Bötsch Y, Jenni L, Kéry M (2020). Field evaluation of abundance estimates under binomial and multinomial N-mixture models. Ibis.

[21] Costa A, Romano A, Salvidio S (2020). Reliability of multinomial N-mixture models for estimating abundance of small terrestrial vertebrates. Biodivers. Conserv..

[22] Ficetola GF (2018). N-mixture models reliably estimate the abundance of small vertebrates. Sci. Rep..

[23] Christensen SA, Farr MT, Williams DM (2021). Assessment and novel application of N-mixture models for aerial surveys of wildlife. Ecosphere.

[24] Couturier T, Cheylan M, Bertolero A, Astruc G, Besnard A (2013). Estimating abundance and population trends when detection is low and highly variable: a comparison of three methods for the Hermann’s tortoise: three methods for estimating the hermanni abundance. J. Wildl. Manag..

[25] Sullivan BL (2009). eBird: a citizen-based bird observation network in the biological sciences. Biol. Cons..

[26] Sullivan BL (2014). The eBird enterprise: an integrated approach to development and application of citizen science. Biol. Cons..

[27] Fink D (2010). Spatiotemporal exploratory models for broad-scale survey data. Ecol. Appl..

[28] Hochachka WM (2012). Data-intensive science applied to broad-scale citizen science. Trends Ecol. Evol..

[29] Johnston A, Moran N, Musgrove A, Fink D, Baillie SR (2020). Estimating species distributions from spatially biased citizen science data. Ecol. Model..

[30] Burnham KP, Anderson DR (2002). Model Selection and Multimodel Inference: A Practical Information-Theoretic Approach.

[31] Strimas-Mackey, M. et al. *Best Practices for Using eBird Data*. Version 1.0 (Cornell Lab of Ornithology, Ithaca, New York, 2020).

[32] Cohen JM, Fink D, Zuckerberg B (2020). Avian responses to extreme weather across functional traits and temporal scales. Glob. Change Biol..

[33] Goldstein, B. R., Turek, D., Ponisio, L. C. & de Valpine, P. nimbleEcology: distributions for ecological models in nimble. (2020).

[34] Wickham H (2016). ggplot2: Elegant Graphics for Data Analysis.

[35] Johnston A (2021). Analytical guidelines to increase the value of community science data: an example using eBird data to estimate species distributions. Divers. Distrib..

[36] R Core Team. R. *A Language and Environment for Statistical Computing* (R Foundation for Statistical Computing, Vienna, Austria, 2020).

[37] Strimas-Mackey, M., Miller, E. & Hochachka, W. Auk: eBird data extraction and processing with AWK. (2018).

[38] Fick S, Hijmans RJ (2017). WorldClim 2: new 1km spatial resolution climate surfaces for global land areas. Int. J. Climatol..

[39] Hijmans, R. J. Raster: geographic data analysis and modeling. (2020).

[40] LANDFIRE. *LANDFIRE Remap 2016 Existing Vegetation Type (EVT) CONUS. Tech. Rep., Earth Resources Observation and Science Center (EROS), U.S. Geological Survey*, (2020).

[41] de Valpine P (2017). Programming with models: writing statistical algorithms for general model structures with NIMBLE. J. Comput. Graph. Stat..

[42] Brooks ME (2017). glmmTMB balances speed and flexibility among packages for zero-inflated generalized linear mixed modeling. R. J..

[43] Hartig, F. DHARMa: residual diagnostics for hierarchical (Multi-Level/Mixed) regression models. (2020).

[44] Johnston A (2019). Analytical guidelines to increase the value of community science data: An example using eBird data to estimate species distributions. Divers. Distrib..

[45] Meehan, T. D., Michel, N. L. & Rue, H. Estimating Animal Abundance with N-Mixture Models Using the R-INLA Package for R. J. Stat. Softw. 10.18637/jss.v095.i02. (2020).

