# Supplementary material for: Comparing N-mixture models and GLMMs for relative abundance estimation in a citizen science dataset
Source: Sci Rep. 2022 Jul 19;12:12276. doi: 10.1038/s41598-022-16368-z (PMC9296480; doi:10.1038/s41598-022-16368-z)
Supplement: Supplementary file 1 — Supplementary Information. [file 41598_2022_16368_MOESM1_ESM.pdf]

Supplemental materials for "Comparing N-mixture models and GLMMs for relative abundance estimation in a citizen science dataset"

Benjamin R. Goldstein and Perry de Valpine

June 3, 2022

1 The N-mixture model: assumptions and sensitivities

The N-mixture model makes the following assumptions:

1. Closure: Each site is assumed to be occupied by a fixed number of individuals across all replicate surveys.

2. No unmodeled heterogeneity in detection probability: Variation among sites in detection probability can be explained by detection covariates.

3. No false positives: If present, individuals might be missed due to imperfect detection. But if absent, they will not be erroneously counted as present.

4. Other: Data are conditionally independent given covariates and site latent variables N; specification of covariate relationships and between-site and within-site distributions is correct.

Assumptions 1-3 are related to the fact that disentangling between- vs within-site variation is possible through the information provided by repeated counts at each site. This means that additional noise in detection probabilities or in the observed counts will bias the decomposition of abundance vs. detection.

Despite filtering the eBird dataset for high-quality checklists, we expect that each of the above assumptions is to some extent violated in our modeling context.

1. It is impossible to assume closure, because even if animals are perfectly stationary, the spatial area covered by observers at a given "site" is unstandardized and likely to vary, resulting in different numbers of individual birds being covered by each sampling event.

2. There is certainly unmodeled heterogeneity in detection probability between eBird surveys due to a variety of unobserved social and environmental factors, such as observer "skill," daily weather, interest or familiarity with different species, use of auditory vs. visual information, etc.

3. There are likely to be some false positives due to misidentifications between similar species or miscounts or rounded counts. eBird includes quality control by monitoring when species appear outside of their known ranges, but it can't know if an observer calls a raven a crow when both occupy an area, or if an observer double-counts a crow in a flock of crows.

2 Species-subregion selection algorithm

To identify target subregions, the complete set of accepted checklists was aggregated to a 500m grid such that each grid cell's value was the number of checklists within 5km of the cell's center. Then the following two-step procedure was iterated: the remaining cell with the greatest value was accepted as the center of a subregion, and all cells within 50 km of that cell were rejected. This was repeated until 20 10 km subregions across California had been identified (Fig. S1).

We considered all species known to breed in California as listed by the North American Breeding Bird Survey (Pardieck et al. 2020). We used the EltonTraits dataset to filter out species that were pelagic specialists (Wilman et al. 2014). Only species detected in at least 20% of checklists in a region qualified for analysis in each subregion. In each region, the 10 species with the highest total number of individuals recorded and the 10 with the lowest total number of individuals recorded (above the 20% threshold) were considered. This yielded 400 species-region model combinations. We also identified the 10 species that qualified in the most regions and accepted their datasets in all regions in which they qualified, in most cases overlapping with those chosen at the region level. Ultimately, 407 species-region datasets were admitted for final analysis. We hypothesized that patterns in model preference might occur along lines of species abundance (e.g. more information supporting more complex models), species identity or functional traits across locations (e.g. beta-binomial detection preference for flocking species), or total number of checklists or sites in the dataset.

3 Fast N-mixture calculations adapted from Meehan et al.

Meehan et al. presented an efficient algorithm for calculating the N-mixture likelihood that took advantage of the recursive properties of the Poisson and binomial distributions. They observed that

$$\text{Pois}(N|\lambda) = \text{Pois}(N-1|\lambda)\frac{\lambda}{N}$$

and

$$\text{Binom}(y|N,p) = \text{Binom}(y|N-1,p)\frac{N}{N-y}(1-p)$$

such that the truncated infinite sum in the N-mixture likelihood can be calculated recursively as

$$\sum_{N=y}^{K} \text{Pois}(N|\lambda)\text{Binom}(y|N,p) = \text{Pois}(y|\lambda)\text{Binom}(y|y,p)(1 + f_1(1 + f_2(1 + f_3(1 + ...))))$$

where

$$f_i = \frac{\lambda(1-p)}{i}$$

We observe that the negative binomial and beta binomial distributions can also be calculated recursively, as:

$$\text{Nbin}(N|r,p) = \text{Nbin}(N-1|r,p)(1-p)(N+r-1)N^{-1}\frac{\lambda}{N}$$

and

$$\text{Betabin}(y|N,\alpha,\beta) = \text{Betabin}(y|N-1,\alpha,\beta)\frac{N(N-1+\beta-y)}{(\alpha+\beta+N-1)(N-y)}$$

These factors were used to develop equivalent recursive calculations for the three additional N-mixture distribution sets (B-NB, BB-P, BB-NB). The recursive strategy is especially useful for the beta binomial cases, where the calculation of beta distributions is the bulk of computation. The rewritten calculation requires only a single call to the beta distribution, resulting in $K-1$ fewer beta calculations. For full code, see the R package nimbleEcology at https://github.com/nimble-dev/nimbleEcology (Goldstein et al., 2020).

We ran compiled versions of the N-mixture likelihood calculation for each N-mixture variation for a range of values of K (the upper bound of the truncated infinite sum) and number of observations. Fast N-mixture calculations led to 20- to 125-fold improvements in computation time relative to calculating the full truncated infinite sum for $100 \leq K \leq 2000$ and $5 \leq \text{length}(y_i) \leq 20$ (Supplemental Figure 5). These gains increase as $\text{length}(y_i)$ increases, such that they will be more important when more replicate visits are made to a site. In eBird, sites are clustered such that a small number of locations contain many replicate observations, making this improvement per replicate particularly relevant.

4 Maximum likelihood estimation in NIMBLE and glmmTMB

NIMBLE is an R package designed for building models, especially hierarchical models (de Valpine et al., 2017). Though it extends BUGS-family MCMC engines, NIMBLE offers additional functionality that enables maximum likelihood estimation (MLE) of arbitrary models. MLE in NIMBLE works as follows. First, a statistical structure is defined in model code. Then, a model object is created and compiled. The model object comprises the complete structure of the directed acyclic graph defined by the model code, and can be used to calculate the likelihood for different parameter values, i.e. the log probability of the data. A simple function is defined that takes values for all model parameters and uses those input values to calculate and return the model likelihood. This function is then passed to a numerical optimizer such as R's 'optim()' function to find the MLE of the parameters. The Hessian matrix approximated by the optimizer is used to estimate standard errors of the parameters under a Gaussian approximation. We implemented N-mixture models in the NIMBLE modeling language for R with custom N-mixture distributions developed for the 'nimbleEcology' auxiliary package (Goldstein et al., 2020). The upper bound of the truncated infinite sum, K, was chosen as the 99.9999th percentile of the Poisson distribution with mean equal to the maximum observed count times 50 (representing 0.02 detection probability), which we expected to be conservative enough to achieve convergence except in the case of estimation failure. For N-mixture models, using a recursive algorithm following Meehan et al. (2018) to calculate the N-mixture likelihood was critical for the computational feasibility of this work (see supplemental section S3).

Maximum likelihood estimation for GLMMs was conducted with the R package "glmmTMB" using Laplace approximation of the likelihood (Brooks et al., 2017).

5 Dropped datasets

In a handful of cases, our GLMM estimator from glmmTMB encountered rounding errors during model optimization of the negative binomial GLMM. This was identified by cases in which the overdispersion parameter was estimated as very large, indicating no overdispersion, but the negative binomial GLMM's AIC was substantially lower than the Poisson GLMM's for the same dataset. In one case, AIC reported by glmmTMB was actually negative. To avoid this issue without implementing ad hoc solutions that could introduce inconsistencies, we excluded 11 datasets from analysis for which the negative binomial overdispersion parameter was estimated above 10^{12}.

6 Difference in N-mixture and GLMM intercepts

The log-scale intercept of the N-mixture model is $\phi_1 = \log(\lambda_0 p_0)$ (log of abundance times detection) while the log-scale intercept of the Poisson GLMM is β_0. Both models are estimating the same quantity (total number of individuals one expects to see), but the mean estimates in practice differ due to differences in model random effect structure (Supplemental Figure 6). Specifically, the log-scale normal random effect of site in the GLMM means that the expected value of the data is $e^{\beta_0 + 0.5\sigma_\alpha^2}$ rather than e^{β_0}. The result is a consistently lower estimate of β_0 relative to ϕ_1. Results in figure 4 instead use parameters adjusted to represent log expected count (rather than expected log count), which for the GLMMs is $\beta_0 + 0.5\sigma_\alpha^2$.

7 Supplemental figures

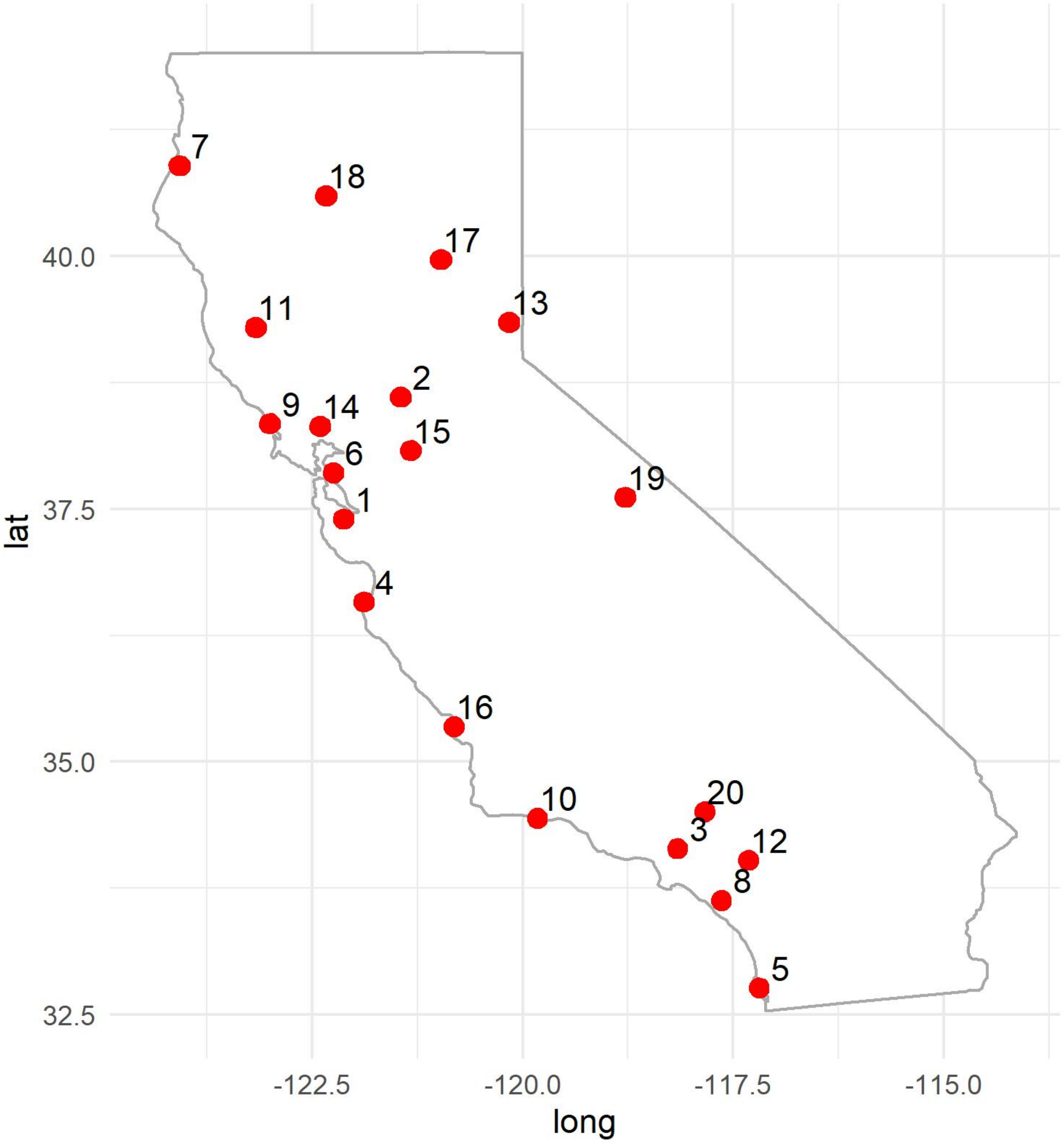

Figure 1: Our site selection algorithm produced twenty subregions representing areas with high intensity of eBird sampling across California. Red circles show the actual size and boundaries of the subregions. Subregion labels are ordered by total number of checklists, with Subregion 1 containing the most checklists.

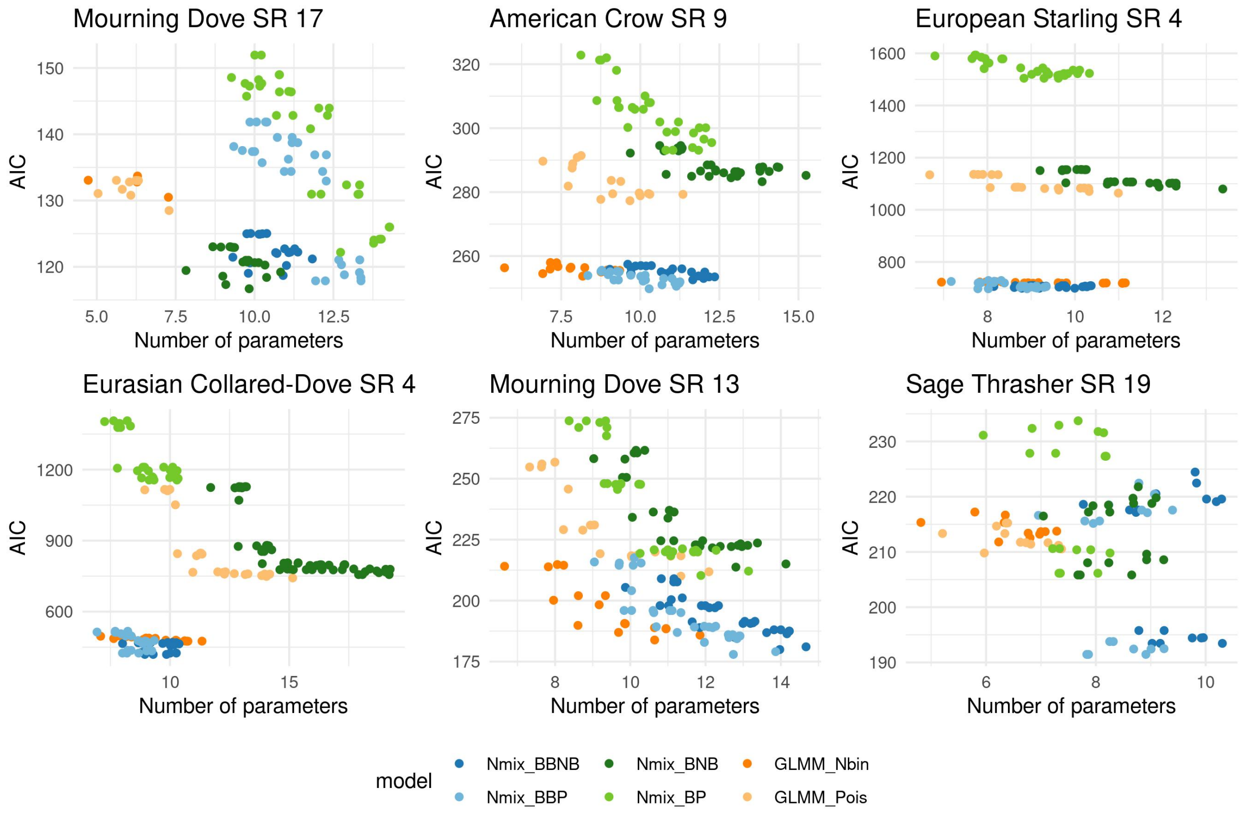

Figure 2: During forward AIC selection, AICs were obtained for many sets of covariates for each model-dataset combination. We plot a few arbitrary example sets of AICs obtained from different numbers of parameters to illustrate this process. Points are jittered horizontally for legibility.

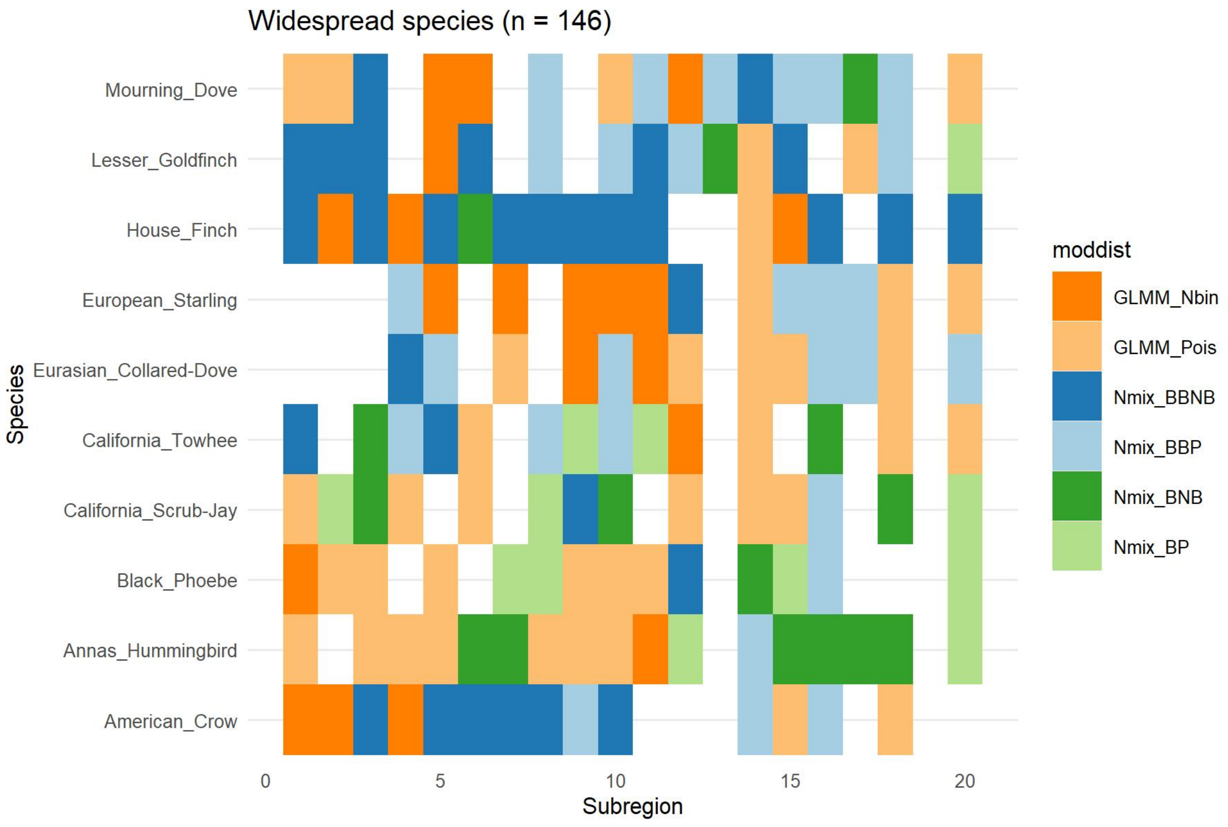

Figure 3: Best model by AIC for each SSRs in the "widespread" category. We did not identify relationships across species.

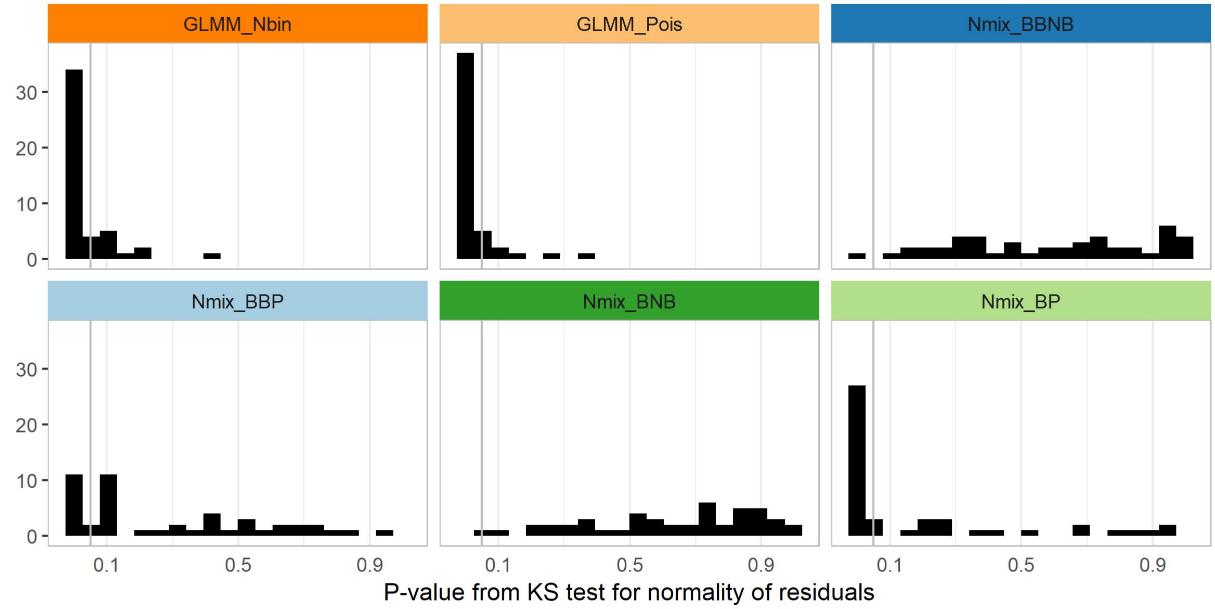

Figure 4: A substantial portion of GLMMs failed goodness-of-fit checks even when they were selected by AIC. In this plot, we isolate all SSRs (1) whose best models by AIC were GLMMs and (2) that failed a goodness-of-fit check ($p < 0.05$) in their best model. We plot histograms of those SSRs' goodness-of-fit p-values across all 6 models. Despite failing goodness-of-fit checks for their "selected" GLMMs, these datasets passed goodness-of-fit for most BB-NB and B-NB N-mixture models.

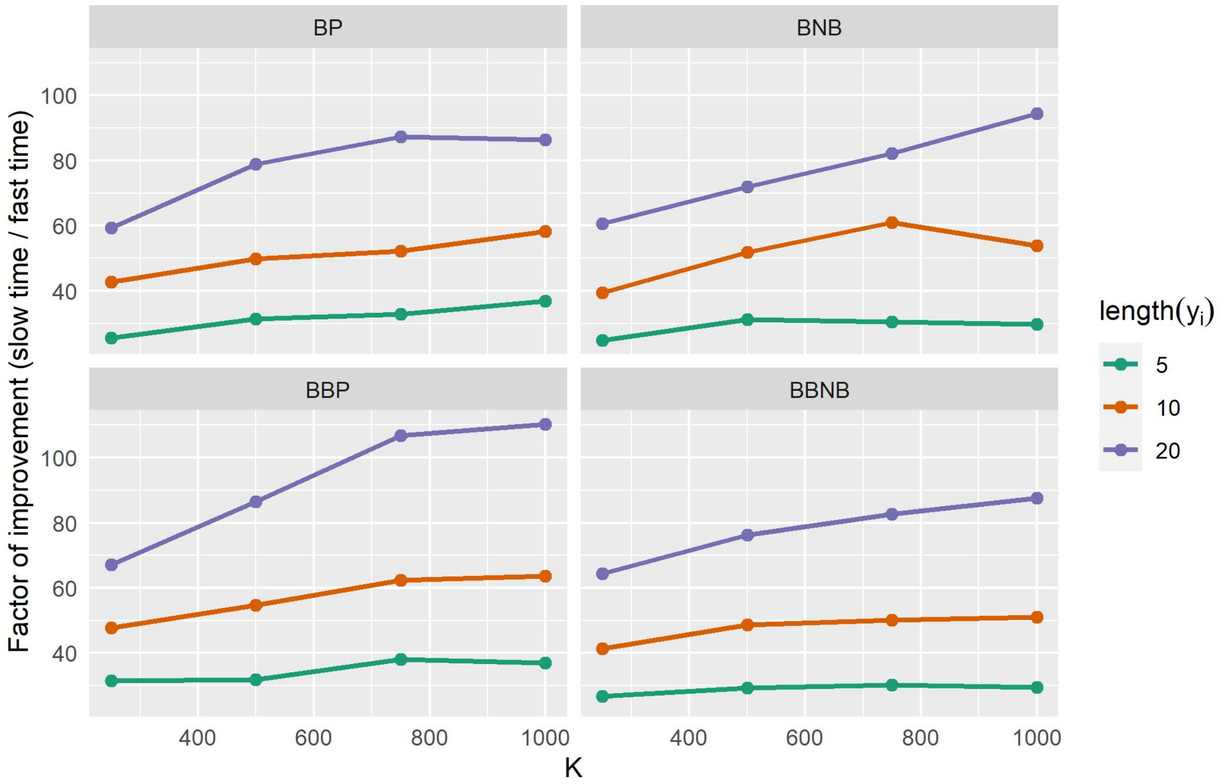

Figure 5: A new implementation of N-mixture model likelihood calculations reduces computation time by a factor of 20-90. We calculated 5,000 N-mixture site likelihoods using the traditional truncated infinite sum and compared the time taken to the same calculation using the fast implementation. Each calculation represents a single site with length(y_i) replicate observations. We varied the upper bound of the infinite truncated sum K and the number of replicate observations. On the y-axis we report the multiplicative improvement in computational time due to the fast N-mixture calculation.

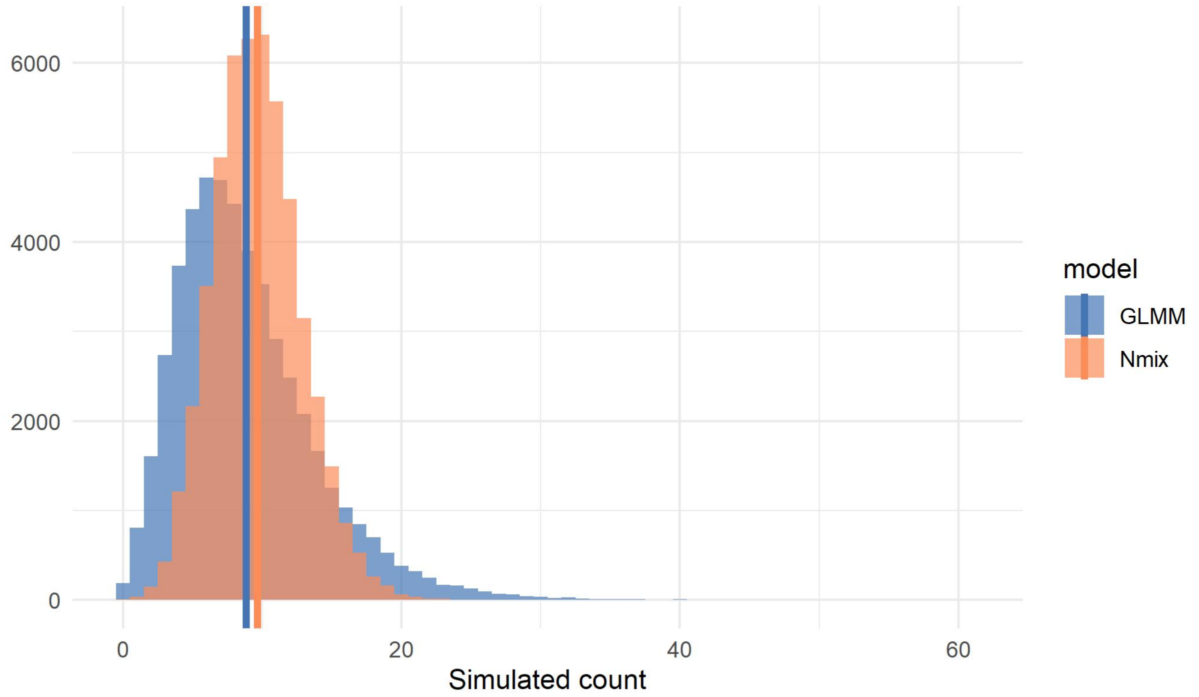

Figure 6: Visualizing the difference in probability distributions for observed counts between the N-mixture model and GLMM. We fit a B-P N-mixture model and a Poisson GLMM with site random effects to a single simulated dataset. We then simulated additional count data from the maximum likelihood parameters for each and show the distribution of counts arising from each fitted model (histograms) and predicted mean counts (vertical lines). This plot illustrates that GLMMs and N-mixture models give different expected counts from the same dataset and reveals how they are estimating meaningfully different models.
